# Inclusion of Older Adults in Consumer-Directed Heat Safety Materials: A Content Analysis of U.S. State Websites

**DOI:** 10.1093/geroni/igaf122.4143

**Published:** 2025-12-31

**Authors:** Christopher Liu, Pierre Jolin, Emily Gadbois, Elizabeth Fussell, Allan Just, Theresa Shireman

**Affiliations:** Brown University, Providence, Rhode Island, United States; Brown University, Providence, Rhode Island, United States; Brown University School of Public Health, Providence, Rhode Island, United States; Institute at Brown for Environment and Society, Providence, Rhode Island, United States; Institute at Brown for Environment and Society, Providence, Rhode Island, United States; Brown University School of Public Health, Providence, Rhode Island, United States

## Abstract

The increasing frequency and duration of extreme heat events places our growing older adult population at heightened risk for adverse, heat-related outcomes. While states provide the public with important heat safety messaging through websites, little is known about the inclusion of targeted information relevant to older adults. We conducted a qualitative content analysis of consumer-directed heat safety information published on state websites to 1) characterize information relevant to older adults and their caregivers and 2) assess information depth and content gaps. Our content analysis revealed five major themes regarding heat-specific risks: 1) acknowledgment of risk in older adults and definitions of older age; 2) chronic conditions or disabilities; 3) use of particular prescription medications or known toxic substances; 4) restricted fluid and salt intake; 5) social isolation. Most websites acknowledged that older adults are at increased risk of heat illness, but only half included a definition for older age. A majority discussed risk in those with chronic conditions—with cardiovascular conditions and obesity included most frequently and cancer and cognitive impairment cited the least. Only half discussed prescription medications, two-thirds discussed toxic substances, and half discussed social isolation as an intersectional concern. Most discussed the importance of consuming fluids and electrolytes, however, few provided recommendations for those with restricted fluid and salt intake. Our results highlight the lack of granular, age-inclusive information in most heat safety material published by state websites. Identified content gaps should inform relevant additions to these materials to promote self-sufficiency in mitigating heat risk among older adults.

